# Absence of histamine-induced itch in the African naked mole-rat and "rescue" by Substance P

**DOI:** 10.1186/1744-8069-6-29

**Published:** 2010-05-24

**Authors:** Ewan St John Smith, Gregory RC Blass, Gary R Lewin, Thomas J Park

**Affiliations:** 1 Dept. of Neuroscience, Max-Delbrück Center for Molecular Medicine, Robert-Rössle-Strasse 10, 13125 Berlin-Buch, Germany; 2 Dept. of Biological Sciences, Univ. of Illinois at Chicago, Chicago, IL, USA

## Abstract

Recent research has proposed a pathway in which sensory neurons expressing the capsaicin activated ion channel TRPV1 are required for histamine-induced itch and subsequent scratching behavior. We examined histamine-induced itch in the African naked mole-rat (*Heterocephalus glaber*) and found that although naked mole-rats display innate scratching behavior, histamine was unable to evoke increased scratching as is observed in most mouse strains. Using calcium imaging, we examined the histamine sensitivity of naked mole-rat dorsal root ganglia (DRG) neurons and identified a population of small diameter neurons activated by histamine, the majority of which are also capsaicin-sensitive. This suggested that naked mole-rat sensory neurons are activated by histamine, but that spinal dorsal horn processing of sensory information is not the same as in other rodents. We have previously shown that naked mole-rats naturally lack substance P (SP) in cutaneous C-fibers, but that the neurokinin-1 receptor is expressed in the superficial spinal cord. This led us to investigate if SP deficiency plays a role in the lack of histamine-induced scratching in this species. After intrathecal administration of SP into the spinal cord we observed robust scratching behavior in response to histamine injection. Our data therefore support a model in which TRPV1-expressing sensory neurons are important for histamine-induced itch. In addition, we demonstrate a requirement for active, SP-induced post-synaptic drive to enable histamine sensitive afferents to drive itch-related behavior in the naked mole-rat. These results illustrate that it is altered dorsal horn connectivity of nociceptors that underlies the lack of itch and pain-related behavior in the naked mole-rat.

## Findings

Itch is an unpleasant sensation that evokes a craving to scratch and is characteristic of many inflammatory skin disorders [1]. Human microneurography studies have identified a subset of mechano-insensitive C-fibers that are strongly activated by histamine and may generate itch [2]. At the molecular level, pharmacological experiments have demonstrated a role for H1-receptors (H_1_R) in histamine-induced scratching in rodents and humans [3,4]. There is also a key role for the capsaicin receptor TRPV1 as TRPV1^-/- ^knockout mice exhibit decreased histamine-induced scratching [5,6]. Centrally, dorsal spinal cord neurons expressing the gastrin-releasing peptide receptor are required for a wide range of pruritogens to evoke itch [7,8]. The neuropeptide substance P (SP) is also implicated in central transmission of itch: intrathecal SP administration evokes scratching [9] and also enhances morphine-evoked scratching [10]. In contrast, deleting the preprotachykinin A gene encoding SP, does not reduce serotonin-evoked scratching behavior [11], thus SP does not mediate all scratching behaviors.

We have previously shown that African naked mole-rats (*Heterocephalus glaber*) do not express SP in small diameter dorsal root ganglia (DRG) neurons [12] and display no nocifensive behavior in response to capsaicin or acid [13]. Intrathecal administration of SP "rescued" a nocifensive licking behavior in response to capsaicin, but did not alter the lack of response to acid, demonstrating that naked mole-rats, uniquely among Mammalia, do not sense acid as a noxious stimulus [14].

**Figure 1:**
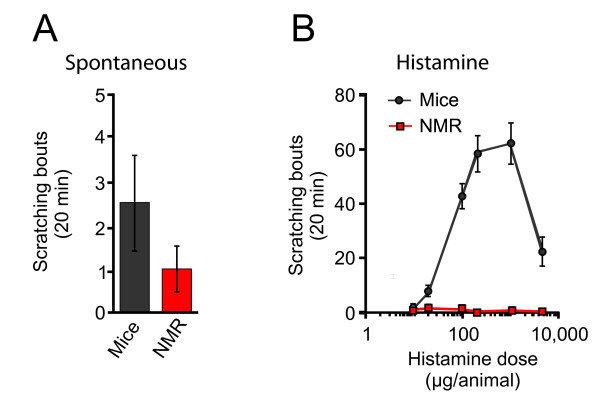
**Spontaneous and histamine-induced scratching in naked mole-rats compared to laboratory mice**. ***A***, Mice and naked mole-rats use their back legs to scratch their bodies at a low spontaneous level. The bar graph shows the average number of bouts of scratching observed over 20 minutes (*n *= 16 mice; 16 naked mole-rats). ***B***, Histamine is not pruritogenic in naked mole-rats. For mice, 10 μg histamine failed to evoke scratching, but higher doses (20 μg - 4.6 mg) evoked pronounced scratching. In contrast, none of the doses tested induced scratching in naked mole-rats.

Considering the importance of TRPV1-expressing C-fibers in histamine-mediated itch [5,6], we decided to investigate whether histamine elicits scratching in naked mole-rats. We initially observed that, at rest, similar to mice, naked mole-rats use their hind legs to scratch their bodies (Fig. 1A). Intracutaneous injection of histamine into the nape of the neck of mice evoked a significant increase in scratching bouts compared to spontaneous levels, peaking at 1000 μg (p < 0.001) and dropping off at a higher dose (4 mg, Fig. 1B). Such non-linear dose-dependency has been previously reported [15]. In stark contrast to mice, naked mole-rats showed no increase in scratching compared to spontaneous levels for any of the histamine doses administered (Fig. 1B).

**Figure 2:**
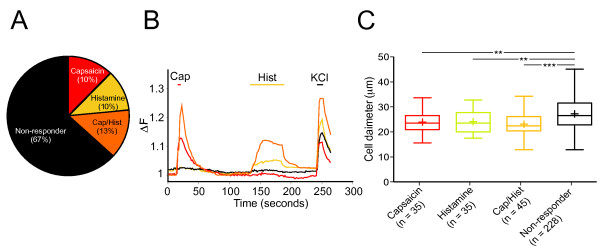
**Histamine activates naked mole-rat DRG neurons**. ***A***, pie-chart showing different populations of DRG neurons, 10% (*n *= 35/343) were activated by 1 μM capsaicin, 10% by 100 μM histamine (*n *= 35/343), 13% were activated by both stimuli (*n *= 45/343). ***B***, example traces showing the time-course of experiments and the different response types. ***C***, groups of neurons responding to either capsaicin, histamine or both were significantly smaller than the group which was not activated by either stimulus (**, p < 0.01 and ***, p < 0.001). Results are taken from 5 different DRG neuron cultures.

The lack of behavioral phenotype could be explained by naked mole-rat sensory neurons being insensitive to histamine and we thus examined this using calcium imaging. Similarly to observations in mice [5], we saw that histamine activates 23.3% of naked mole-rat DRG neurons, evoking slowly activating calcium responses (*n *= 80/343, Fig. 2A and 2B). Of these histamine-sensitive neurons, 56.3% were also capsaicin-sensitive (45/80) thus identifying the majority as C-fibers, which concurs with human data showing that histamine "itch" C-fibers are commonly capsaicin-sensitive [2]. Furthermore, capsaicin-, histamine- and histamine/capsaicin-sensitive neurons were all significantly smaller than those not responding to either stimulus, suggestive of a C-fiber phenotype (22.2 ± 0.8 μm, p < 0.01; 22.4 ± 0.76 μm, p < 0.01; 21.3 ± 0.6 μm, p < 0.001 versus 25.5 ± 0.4 μm respectively, see Fig. 2C). The histamine-sensitive, capsaicin-insensitive DRG neurons exhibited significantly smaller histamine-evoked calcium responses compared to histamine/capsaicin-sensitive neurons (0.103 ± 0.01 ΔF vs. 0.155 ± 0.02 ΔF *n *= 35 and 38 respectively, p < 0.05). To examine if TRPV1 played a part in amplifying histamine-evoked responses in the capsaicin-positive population (as has been observed in the mouse [5]), experiments were repeated where histamine was applied in the presence of the TRP channel blocker ruthenium red (RR, 10 μM). Histamine-evoked calcium responses were smaller in the presence of RR, but not significantly so (0.119 ± 0.01 ΔF vs 0.155 ± 0.02 ΔF *n *= 29 and 38 respectively, p = 0.188), suggesting that amplification of histamine-induced calcium influx by TRPV1 only plays a minor role, if any, in the naked mole-rat. With their smaller responses to histamine, the histamine-sensitive, capsaicin-insensitive population may correspond to mechanosensitive C-fibers in humans that are only weakly excited by histamine with a time-course, which does not match that of histamine itch sensation [2]. However, a role in non-histamine itch for the histamine-sensitive, capsaicin-insensitive C-fibers in naked mole-rats cannot be ruled out because in some species all mechanosensitive C-fibers are activated by the pruritogen cowhage [16].

**Figure 3:**
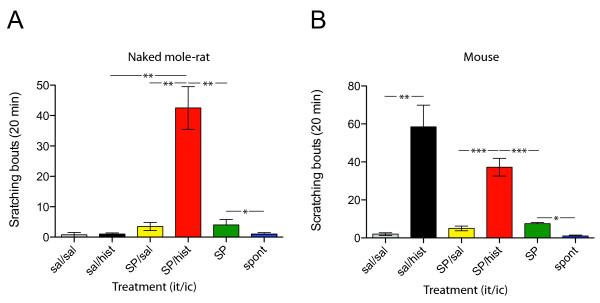
**Substance P "rescues" histamine-mediated scratching in naked mole-rats**. ***A***, naked mole-rats: after intrathecal administration of saline, intracutaneous histamine fails to evoke any more scratching than saline (black bar vs. grey bar), however after intrathecal SP treatment, histamine evokes robust scratching (red bar). Intrathecal administration of SP alone (green bar) increases scratching compared to spontaneous levels (blue bar), but combining intrathecal SP with subsequent intracutaneous saline induces no greater effect (yellow bar). ***B***, mice: intracutaneous histamine causes increased scratching (black bar) compared to saline alone (grey bar) and is of a similar magnitude following intrathecal SP treatment (red bar). Intrathecal SP administration induces more scratching than is observed spontaneously (green vs. blue bar), but combining intrathecal SP with subsequent intracutaneous saline induces no greater effect (yellow bar). *N *= 4 animals for each group; it/ic, intrathecal treatment/intracutaneous treatment; sal, saline, hist, histamine, SP, substance P, spont, spontaneous and *, p < 0.05, **, p < 0.01 and ***, p < 0.001.

We have previously shown that the lack of nocifensive response to capsaicin in naked mole-rats is "rescued" by intrathecal administration of SP, a neuropeptide that their cutaneous C-fibers lack [13]. We therefore tested if intrathecal administration of SP affected naked mole-rat responses to histamine. Remarkably, following intrathecal SP administration, naked mole-rats displayed robust scratching behavior in response to histamine that was significantly greater than after intrathecal saline (42.5 ± 7.0 vs. 1.0 ± 0.4 bouts/20 min, p < 0.01 Fig. 3A), reaching similar levels to that observed in mice (Fig. 3B). To be certain that the effect observed in naked mole-rats was specific to histamine, we examined the effect of intracutaneous saline following intrathecal SP administration. This resulted in scratching levels that were not significantly higher than when intrathecal SP was given alone (3.5 ± 1.3 vs 4.0 ± 1.8 bouts/20 min Fig. 3A) thus confirming that the effect of histamine was specific and not due to the noxious nature of the injection procedure itself. Similarly in mice, intracutaneous saline after intrathecal SP administration did not result in a larger number of scratching bouts than was observed after intrathecal SP administration alone (Fig. 3B). Intrathecal injection of SP alone has been reported previously to induce scratching, biting, and licking in laboratory rodents [9,17]. In this study, the number of bouts of scratching from SP alone was in fact significantly greater than spontaneous levels for both species (naked mole-rats = 4.0 ± 1.8 vs 0.9 ± 0.05 bouts/20 min, p < 0.05 Fig. 3A; mice = 7.5 ± 0.7 vs 2.6 ± 1.1 bouts/20 min, p < 0.05, Fig. 3B). Furthermore, in both species the administration of histamine, following intrathecal SP, evoked significantly greater levels of scratching than SP alone (NMR: 42.5 ± 7.0 vs 4.0 ± 1.8 bouts/20 min, p < 0.01, Fig. 3A and mouse: 37.3 ± 4.7 vs 7.5 ± 0.6 bouts/20 min, p < 0.001, Fig. 3B).

We also observed an interesting behavior in naked mole-rats in response to histamine after intrathecal SP administration. In addition to episodic scratching behavior (additional file 1), naked mole-rats spent short periods of time (~5 seconds) vigorously rubbing their backs against the walls of the observation chamber (additional file 2), a behavior that was novel to us as experienced observers of naked mole-rat behavior and did not occur when intracutaneous saline was administered following intrathecal SP.

We have previously hypothesized that capsaicin fails to evoke nocifensive behavior in naked mole-rats because unlike in the mouse, where C-fibers predominantly terminate in the superficial "pain-signaling" laminae of the spinal cord dorsal horn, naked mole-rat C-fibers terminate in superficial and deeper laminae equally [13]. Reintroduction of SP, via intrathecal administration, "rescued" behavioral sensitivity to capsaicin. We propose that addition of SP activates superficially located neurons expressing neurokinin-1 receptors and thus increases the excitatory drive into the superficial laminae, which overrides the simultaneous activation of deep dorsal horn neurons, which is unique to the naked-mole-rat.

In this study we find that SP is also able to "rescue" histamine-mediated scratching behavior, as well as inducing a vigorous back-rubbing behavior. This "rescue" suggests that naked mole-rat primary afferent neurons are normally activated by histamine, which our calcium imaging results confirm (Fig. 2). We suggest therefore that peripheral activation of naked mole-rat C-fibers by histamine usually leads to both superficial and deeper spinal laminae being activated and that SP administration allows for information travelling via superficial pathways, known to be involved in itch processing [18], to dominate, thus leading to the expression of scratching behavior. Because of the observation that SP administration enables histamine pruritogenicity in naked mole-rats, we suggest that a portion of the same C-fiber population that is responsible for transmitting capsaicin "pain" information may also transmit histamine "itch" information. Consequently, these results also support the model that TRPV1 expressing neurons are required for histamine-mediated scratching [5,6].

Our data are also supportive of a role for SP in scratching behavior. Like others, we found that SP administration induces scratching [9], in addition to the "rescuing" of histamine-induced scratching behavior in naked mole-rats. However, it should be noted that histamine is relatively non-pruritinogenic in certain laboratory rodent strains, which endogenously express SP (ddY mice [19] and Sprague-Dawley rats [20]). Therefore, the importance of SP in mediating histamine-mediated scratching could very well be species/strain dependent, but clearly plays a key role in the naked mole-rat.

We can only speculate as to why naked mole-rats have evolved insensitivity to the pruritogenic effects of histamine. One possible explanation is that histamine-insensitivity is a result of sensory nervous system adaptations, which have evolved under selection pressure unrelated to itch. In the wild, naked mole-rats live in large colonies underground, which results in unusually high levels of carbon dioxide and ammonia [21]. Carbon dioxide can be converted into carbonic acid, which activates C-fiber nociceptors causing pain [22-24] and the ability to detect acid as a noxious stimulus is evolutionarily conserved amongst both invertebrates and vertebrates [14]. Therefore, the finding that naked mole-rats do not demonstrate any nocifensive response to acid suggests that the sensory nervous system of naked mole-rats (and their ancestors) has evolved under the selection pressure of noxiously high levels of environmental carbon dioxide with the result that acid is no longer detected as noxious [13]. A lack of behavioral avoidance to ammonia [25] and loss of neuropeptides in cutaneous C-fibers [12] are also adaptations that may well have evolved in response to the unusual atmosphere that ancestors of the naked mole-rat experienced in order to alter how noxious stimuli are processed by the nervous system. Although this is only one possible explanation for how these unusual features of the naked mole-rat sensory nervous system have evolved, the histamine-insensitivity documented in this study may have arisen as a by-product of this evolutionary process. Indeed, the lack of histamine sensitivity is likely to be related to the lack of behavioral sensitivity to capsaicin that we have already documented and which is probably explained by the unusual connectivity of capsaicin-sensitive C-fibers in this species.

In summary, we show that the African naked mole-rat lacks histamine-mediated itch-related behavior, even though capsaicin-sensitive C-fibers are commonly histamine-sensitive in this species. However, histamine-induced scratching can be "rescued" after intrathecal SP administration. Our data support the theory that a population of TRPV1-positive C-fibers mediate histamine-induced itch and that it is the unique connectivity of these neurons in the spinal cord of naked mole-rats that underlies the absence of itch.

## Materials and methods

### Animals

In this study we tested 2-3 month-old, male C57BL6 mice (mean body weight = 21.7 g) and 6-18 month-old naked mole-rats (mean body weight = 20.0 g). The maximum life span of naked mole-rats approaches 30 years and those used here were considered to be young adults. Animal protocols were approved by the University of Illinois at Chicago Institutional Animal Care and Use Committee or German federal authorities (State of Berlin) as appropriate.

### Histamine and scratching behavior

Histamine (Sigma) dissolved in physiological saline was administered by intracutaneous injection between the shoulder blades. Histamine concentrations and injection volumes were generally based on those reported by Green, et al. [15]. Doses ranged from 10 μg/animal (0.65 mM) to 4.6 mg/animal (300 mM), delivered in a volume of 50 μl; doses encompassing those shown to be effective across a variety of mouse strains [15]. After injection of histamine, we observed an animal for 20 minutes, during which time we counted bouts of scratching, which is referred to in the text as bouts/20 minutes. A bout of scratching was defined as the animal lifting a hind paw from the cage floor, scratching at the injection site with the paw, and returning the paw to the floor. Animals were tested in their home cages. Statistical comparisons between the mean scratching bouts evoked by different histamine doses were made using Student's T test.

### Intrathecal application of SP

Animals were briefly anesthetized (~1-2 min) with isoflurane (2% in 2 L/min O_2_). Substance P, dissolved in physiological saline (100 μM in a 20 μl volume) was administered intrathecally between vertebrae L4 and L5. After recovery from anesthesia (~5 min later), histamine was applied and scratching behaviors recorded. The observer, counting the bouts of scratching, was blind to the whether saline or SP was administered intrathecally and to whether saline or histamine was administered intracutaneously. Statistical comparisons between the means were made using Student's T test.

### Calcium imaging

DRG neurons from all spinal levels were prepared from naked mole-rat as described previously [13] and plated on to poly-L-lysine (200 mg/ml) and laminin (20 μg/ml) coated glass coverslips. Neurons were maintained in DMEM (Life Technologies) containing 10% heat-inactivated horse serum (Biochrom), 20 mM glutamine, 0.8% glucose, 100 U penicillin and 100 mg/ml streptomycin (Life Technologies) and incubated at 37°C in 5% CO_2_. Standard Fura-2 ratiometric calcium imaging was conducted to measure responses to histamine (100 μM, 60 seconds) and capsaicin (1 μM, 5 seconds). At the end of each experiment KCl (40 mM, 10 seconds) was administered to verify that cells were healthy neurons. In some experiments ruthenium red (10 μM, 10) was added to the histamine solution. An inverted microscope (Zeiss Observer A1) equipped with MetaFluor photonics imaging system, including a Polychromator V, and a CoolSNAP ES camera (Visitron) was used for cell imaging. Cell soma diameters were measured and the mean values for different groups are given ± the standard error of the mean (S.E.M.). Differences between groups, in terms of diameter and the magnitude of calcium responses, were assessed using Students T-test,

## Competing interests

The authors declare that they have no competing interests.

## Authors' contributions

TJP conceived the study with all authors being responsible for experimental design. EStJS conducted calcium-imaging experiments. GRCB and TJP carried out behavioral experiments. EStJS, GRL and TJP wrote the manuscript, the final version of which has been read and approved by all authors.

## Supplementary Material

Additional file 1 Naked mole-rats display low-level spontaneous scratching with the hind paws, similar to mice. Click here for file

Additional file 2 In addition to scratching, naked mole-rats display bouts of vigorous back rubbing against the cage wall after combined intrathecal application of SP and intracutaneous histamine. Presumably the back rubbing is an attempt to scratch its back. Click here for file
